# Fructose‐1,6‐bisphosphatase aggravates oxidative stress‐induced apoptosis in asthma by suppressing the Nrf2 pathway

**DOI:** 10.1111/jcmm.16439

**Published:** 2021-05-07

**Authors:** Jiapeng Hu, Jia Wang, Chunlu Li, Yunxiao Shang

**Affiliations:** ^1^ Department of Pediatrics Shengjing Hospital of China Medical University Shenyang China

**Keywords:** apoptosis, asthma, Fbp1, oxidative stress

## Abstract

Asthma is a chronic airway disease that causes excessive inflammation, oxidative stress, mucus production and bronchial epithelial cell apoptosis. Fructose‐1,6‐bisphosphatase (Fbp1) is one of the rate‐limiting enzymes in gluconeogenesis and plays a critical role in several cancers. However, its role in inflammatory diseases, such as asthma, is unclear. Here, we examined the expression, function and mechanism of action of Fbp1 in asthma. Gene Expression Omnibus (GEO) data sets revealed that Fbp1 was overexpressed in a murine model of asthma and in interleukin (IL)‐4‐ or IL‐13‐stimulated bronchial epithelial cells. We confirmed the findings in an animal model as well as Beas‐2B and 16HBE cells. In vitro investigations revealed that silencing of Fbp1 reduced apoptosis and the proportion of cells in the G2/M phase, whereas overexpression led to increases. Fbp1 knock‐down inhibited oxidative stress by activating the nuclear factor erythroid 2‐related factor 2 (Nrf2) pathway, whereas Fbp1 overexpression aggravated oxidative stress by suppressingthe Nrf2 pathway. Moreover, the Nrf2 pathway inhibitor ML385 reversed the changes caused by Fbp1 inhibition in Beas‐2B and 16HBE cells. Collectively, our data indicate that Fbp1 aggravates oxidative stress‐induced apoptosis by suppressing Nrf2 signalling, substantiating its potential as a novel therapeutic target in asthma.

## 1 INTRODUCTION

Bronchial asthma is a common chronic disease of the airway, with clinical signs that include dyspnoea, chest tightness, wheezing and coughing. Asthma is a major public health concern and affects approximately 334 million individuals worldwide, with evidence of an increasing prevalence.^1^ Allergic asthma is characterized by Th2‐mediated inflammation, typically initiated by antigen‐presenting and bronchial epithelial cells.^2^ Previous studies have shown that bronchial epithelial injury is a cardinal component of asthma pathogenesis and is correlated with disease severity.^3^ As asthma progresses, bronchial epithelial cells undergo aberrant apoptosis under allergen challenge. An increased rate of apoptosis has been reported in patients with severe asthma.^4^ The delayed or defective clearance of apoptotic cells in individuals with asthma exacerbates airway inflammation and airway hyper‐responsiveness (AHR).^5^ Additionally, the occurrence of oxidative stress caused by an imbalance between oxidation and antioxidation plays a critical role in asthma via activation of inflammatory signalling and bronchial epithelial cell apoptosis.^6^, ^7^ However, the apoptosis and oxidative stress mechanisms of bronchial epithelial cells in asthma are largely uncharacterized.

Fructose‐1,6‐bisphosphatase (Fbp1) is a rate‐limiting enzyme of gluconeogenesis that catalyses the splitting of fructose‐1,6‐bisphosphate into fructose 6‐phosphate and inorganic phosphate.^8^ Fbp1 reportedly plays a critical role in several diseases, including type 2 diabetes mellitus,^9^ cancers^10^, ^11^, ^12^ and acute liver failure.^13^ Kaur et al^9^ reported that Fbp1 can be considered a potential target for the treatment of type 2 diabetes. In cholangiocarcinoma cells, overexpression of Fbp1 induces apoptosis and suppresses cell proliferation, migration and invasion.^14^ Loss of Fbp1 also represses reactive oxygen species (ROS) production in breast cancer.^15^ Wang et al^13^ suggested that Fbp1 is a promising biomarker of acute liver failure, as high serum levels of Fbp1 were found to be correlated with high mortality. However, the role of Fbp1 in inflammatory diseases, such as asthma, has not been elucidated.

Studies have shown that nuclear factor erythroid‐derived 2‐related factor 2 (Nrf2) is an important endogenous antioxidant and anti‐apoptotic transcription factor. The Nrf2 pathway is considered a vital mechanism of protection in individuals with asthma, and Nrf2 signalling is crucial for maintaining bronchial epithelial barrier integrity.^16^ Moreover, Nrf2 activators have been shown to ameliorate airway inflammation, AHR and oxidative stress in mice.^17^, ^18^ However, the relationship between Fbp1 and the Nrf2 pathway has not been identified.

In the current study, we aimed to investigate the expression of Fbp1 in a murine model of asthma and interleukin (IL)‐4‐stimulated or IL‐13‐stimulated airway bronchial cell lines. Furthermore, we sought to examine the mechanisms underlying the effects of Fbp1 on oxidative stress‐induced apoptosis in asthma.

## 2 MATERIALS AND METHODS

### 2.1 Bioinformatics analyses

We downloaded seven gene expression data sets associated with asthma from the Gene Expression Omnibus (GEO) database (https://www.ncbi.nlm.nih.gov/geo/). The GSE41667, GSE6858, GSE41665 and GSE79156 data sets were relevant to the murine model of ovalbumin‐induced asthma, and the GSE19182, GSE37693 and GSE78914 data sets were relevant to IL‐4‐stimulated or IL‐13‐stimulated bronchial epithelial cells. The GSE19182 data set was divided into two data sets because both IL‐4 and IL‐13 were used to stimulate the bronchial epithelial cells. Data pre‐processing was carried out using a robust multi‐array average derived using the Affy package in R (v3.5.1; http://www.r‐project.prg/).^19^ Differentially expressed genes (DEGs) were identified using the limma package in R.^20^ GEO2R (https://www.ncbi.nlm.nih.gov/geo/geo2r/) was also used to screen for DEGs in the asthmatic and control groups.^21^ Significant DEGs were selected using the criteria of |log fold change| > 1 and *P* < .05 and visualized as volcano plots constructed using the R package ggplot2.^22^ Additional DEG data for the asthmatic mouse and control groups were selected from a previous study.^23^ A plot of the overlapping up‐regulated gene sets was created using the R package UpSetR.^24^ The overlapping gene expression of Fbp1 in each data set was presented as a bar graph.

### 2.2 Murine model of ovalbumin‐induced asthma

Female BALB/c mice, 6‐7 weeks old (weight 18‐19 g), were purchased from Beijing Hfk Bioscience Co., Ltd and housed for 7 days to acclimate to the laboratory environment. All mice were housed under standard pathogen‐free laboratory conditions with a 12‐hour dark/light cycle, temperature of 22 ± 2°C and humidity of 55 ± 5% and provided ad libitum access to food and water. All animal experiments were conducted in accordance with the Chinese National Regulations for Animal Care and the guidelines of the China Medical University Animal Care and use Committee, Shenyang, China (approval no. 2020PS665K).

The mice were sensitized with 50 µg ovalbumin (OVA; Grade V, Sigma‐Aldrich) mixed with 0.8 mg aluminium hydroxide (Imject Alum Adjuvant, Thermo Fisher Scientific) in sterile saline by intraperitoneal (i.p.) injection on days 0, 7 and 14, as described previously.^25^ On days 21‐28, the mice were challenged for 30 minutes with 2% OVA using a nebulizer (DeVilbiss). Control mice were treated according to the same protocol but received saline only in the sensitization and challenge phases.

### 2.3 Histological examination of lung tissue

Lung tissue specimens were fixed in 4% paraformaldehyde for 48 hours, embedded in paraffin blocks and sliced into 4‐μm‐thick sections. The sections were stained with haematoxylin and eosin (HE; Solarbio) to detect inflammatory cell infiltration or Periodic acid‐Schiff (PAS; Wanleibio Group, Inc) to measure mucus production in the goblet cells.

### 2.4 Bronchoalveolar lavage fluid (BALF) and cell counts

Mice were sacrificed by i.p. injection of overdose sodium pentobarbital 24 hours post‐challenge, and BALF was collected as previously described.^26^ Briefly, mouse tracheae were intubated with a catheter after the ligation of the right main bronchus, and BALF was collected by rinsing the left lung three times with 0.5 mL standard saline. The BALF was centrifuged (188 *g* , 8 minutes, 4°C) and the supernatant was collected for cytokine and chemokine detection. The cell pellets were resuspended in 0.5 mL standard saline and prepared for cell quantification and morphological analysis using Wright‐Giemsa staining (Solarbio). Two researchers independently counted and typed a minimum of 200 cells/sample.

### 2.5 Immunohistochemistry

After fixation in 4% formalin and subsequent embedding in paraffin, lung tissues were sectioned at a 3‐μm thickness and stained with anti‐Fbp1 antibody (1:200, Abcam). ImageJ software (National Institutes of Health) was used to assess protein expression.

### 2.6 Cell culture transfection and treatment

The human bronchial epithelial cell line Beas‐2B was purchased from Conservation Genetics CAS Kunming Cell Bank and cultured in Beas‐2B cell‐specific serum‐free medium (Conservation Genetics CAS Kunming Cell Bank). The human bronchial epithelial cell line 16HBE was purchased from the Xiangya School of Medicine and cultured in RPMI‐1640 medium (Bioind) supplemented with 10% foetal bovine serum (Bioind). Both cell lines were maintained in a humidified incubator with 5% (v/v) CO_2_ at 37°C. To mimic the Th2 environment of asthma, cells were treated with human recombinant IL‐4 (20 ng/mL) or IL‐13 (20 ng/mL) (Peprotech, #200‐04 and #200‐13, respectively). The cells were transfected with small interfering (si)RNA to form the Fbp1‐silencing group (Fbp1‐si group) and siRNA‐negative control group (Si‐nc group). Fbp1 siRNA1 (CGACCTGGTTATGAACATGTT) and Fbp1 siRNA2 (AGCAGTCAAAGCCATCTCTT) were synthesized by GenePharma Co., Ltd. To generate the Fbp1 overexpression group (Fbp1 group) and plasmid negative control group (P‐nc group), the cells were transfected with either Fbp1 plasmid, which encoded full‐length Fbp1 cDNA, or an empty vector, which served as the negative control, respectively (Hanbio Biotechnology Co., Ltd). Transfections were performed using TransIntro EL Transfection Reagent (TransGen Biotech) according to the manufacturer's instructions. The Nrf2 inhibitor ML385 (5 μmol/L, Apexbio) was simultaneously added to the cells being transfected with Fbp1 siRNA or negative control siRNA. Forty‐eight hours post‐transfection, the cells were stimulated with IL‐13 (20 ng/mL).^25^, ^27^ Detection of the indicated markers was performed 48 hours post‐IL‐13 treatment.

### 2.7 Western blot analysis

Lung samples and culture cells were homogenized with cold radioimmunoprecipitation buffer (Beyotime Biotechnology) together with protease inhibitor (Meilunbio) and phenylmethanesulfonyl fluoride (Beyotime Biotechnology). Nuclear protein was extracted from cells using a nuclear protein extraction kit according to the manufacturer's instructions (Beyotime Biotechnology). Protein concentration was measured using an enhanced BCA protein assay kit (Beyotime Biotechnology). Protein samples (25 μg) were electrophoresed and separated on 10% SDS polyacrylamide gels and then transferred to polyvinylidene fluoride membranes. The membranes were blocked in 5% skim milk or 5% bovine serum albumin (BSA) at room temperature for 2 hours and then incubated with primary antibodies at 4°C overnight. Antibodies against Nrf2 (ab62352, 1:5000), HO‐1 (ab68477, 1:5000), caspase/cleaved‐caspase‐3 (ab32351, 1:1000), and Fbp1 (ab109732, 1:5000), p‐Nrf2 (ab76026, 1:5000) were purchased from Abcam. Antibodies against Bax (5023, 1:1000), Keap1 (8047, 1:1000), p‐Stat6 (56554, 1:1000) and Histone H3 (4499, 1:2000) were purchased from Cell Signaling Technology. Antibodies against Bcl‐2 (60178‐1‐Ig, 1:5000), Stat6 (66717‐1‐Ig, 1:5000), E‐cadherin (20874‐1‐AP, 1:5000), N‐cadherin (22018‐1‐AP, 1:5000), Vimentin (10366‐1‐AP, 1:5000), α‐SMA (14395‐1‐AP, 1:4000) and β‐actin (66009‐1‐Ig, 1:10 000) were purchased from Proteintech Group, Inc. The membranes were washed and then incubated with the appropriate horseradish peroxide‐conjugated secondary antibodies (ZSGB‐BIO) for 2 hours at room temperature before being treated with enhanced chemiluminescent reagent (Wanleibio Group, Inc). The chemiluminescent signals were detected using an Amersham Imager 600 (GE Healthcare Life Sciences). Grey values were determined using ImageJ software. The experiments were independently repeated at least three times.

### 2.8 RNA preparation and quantitative real‐time polymerase chain reaction (qPCR)

Total RNA was extracted from lung tissues and cells using TRIzol Reagent (Takara Bio). Absorbance values at 260/280 nm were measured with a NanoDrop spectrophotometer (Thermo Fisher Scientific) and used to calculate RNA quantity and purity. Complementary DNA (cDNA) was generated using the extracted RNA and a cDNA Synthesis Kit (Takara Bio) and amplified using a LightCycler 480 (Roche Molecular Systems, Inc) and SYBR PrimeScript RT‐PCR Kit (Takara Bio). The sequences of the mouse Fbp1 PCR primers were 5′‐AGTCGTCCTACGCTACCTGTG‐3′ (forward) and 5′‐GGGGATCGAAACAGACAACAT‐3′ (reverse). The sequences of mouse β‐actin PCR primer were 5′‐GTGCTATGTTGCTCTAGACTTCG‐3′ (forward) and 5′‐ATGCCACAGGATTCCATACC‐3′ (reverse). The 2^−ΔΔct^ method was used to assess the relative mRNA fold changes with β‐actin serving as the reference gene. All data shown represent the average results from three replicates.

### 2.9 ROS production analysis

Beas‐2B and 16HBE cells were stimulated with IL‐13 for 48 hours. The cells were then incubated with 20 µM 2′,7′‐dichlorofluorescin diacetate (Sigma) for 30 minutes. ROS expression was evaluated using a Multi‐Mode Microplate Reader (BioTek Synergy HT) and fluorescence microscope (Olympus).

### 2.10 Oxidative stress and antioxidant enzyme analysis

Cellular malondialdehyde (MDA), superoxide dismutase (SOD) and glutathione (GSH) levels were quantified using an MDA Assay Kit (Beyotime), GSH Assay Kit (Solarbio) and total‐SOD Assay Kit (Elabscience), respectively, according to the manufacturers' instructions. Assay results were measured using a Multi‐Mode Microplate Reader (BioTek Synergy HT). All data were normalized to protein concentrations.

### 2.11 Cell cycle assay

Cells were harvested and fixed in 70% ethanol for 12 hours at −20°C. The cells were then treated with RNase A (20 mg/mL) for 30 minutes at 37°C followed by treatment with 50 mg/mL propidium iodide (Solarbio) for 30 minutes in the dark at room temperature. The cell cycle was assessed by flow cytometry using a FACSCalibur (BD Biosciences).

### 2.12 Cell apoptosis assay

Apoptosis was evaluated using an Annexin‐V‐PE/7‐AAD Apoptosis Detection Kit (Vazyme). Briefly, cells were harvested with trypsin, washed with cold phosphate buffered saline (PBS), resuspended in 500 μL binding buffer and incubated with 5 μL Annexin‐V and 5 μL PE for 20 minutes in the dark. The fluorescence signal was analysed by flow cytometry (Guava Technologies Inc).

### 2.13 Statistical analysis

All data are expressed as the mean ± standard deviation. A *t* test was performed when assessing two groups, while one‐way analysis of variance (ANOVA) was used for comparing multiple groups from a minimum of three independent experiments. SPSS 22.0 software (SPSS) and GraphPad Prism software Version 8.0 (GraphPad Software) were used to perform statistical analysis. *P* < .05 was considered statistically significant.

## 3 RESULTS

### 3.1 Fbp1 expression levels in asthma were up‐regulated based on bioinformatics analyses

The GEO database was used to examine DEGs in the asthmatic and control groups. Ovalbumin is commonly employed in murine models of asthma for sensitization and challenge.^28^ Accordingly, we selected four GEO data sets of the murine model of ovalbumin‐induced asthma to identify the core genes in asthma: GSE6858, GSE41665, GSE41667 and GSE79156. In addition to these four data sets, another data set was obtained from a previously published study that used microarray to evaluate DEGs in asthmatic mice compared to those in control mice.^23^ Furthermore, because allergic asthma is considered a Th2‐type disease, Th2 cytokines such as IL‐4 and IL‐13 are widely applied in vitro to mimic the asthmatic environment.^29^, ^30^ In the current study, we selected three IL‐4‐stimulated or IL‐13‐stimulated bronchial epithelial cell GEO data sets: GSE19182, GSE37693 and GSE78914. As both IL‐4 and IL‐13 were used in data set GSE19182, we performed separate analyses regarding IL‐4 and IL‐13 for this database. Overall, a total of eight GEO data sets and one previously published data set were selected.

**FIGURE 1:**
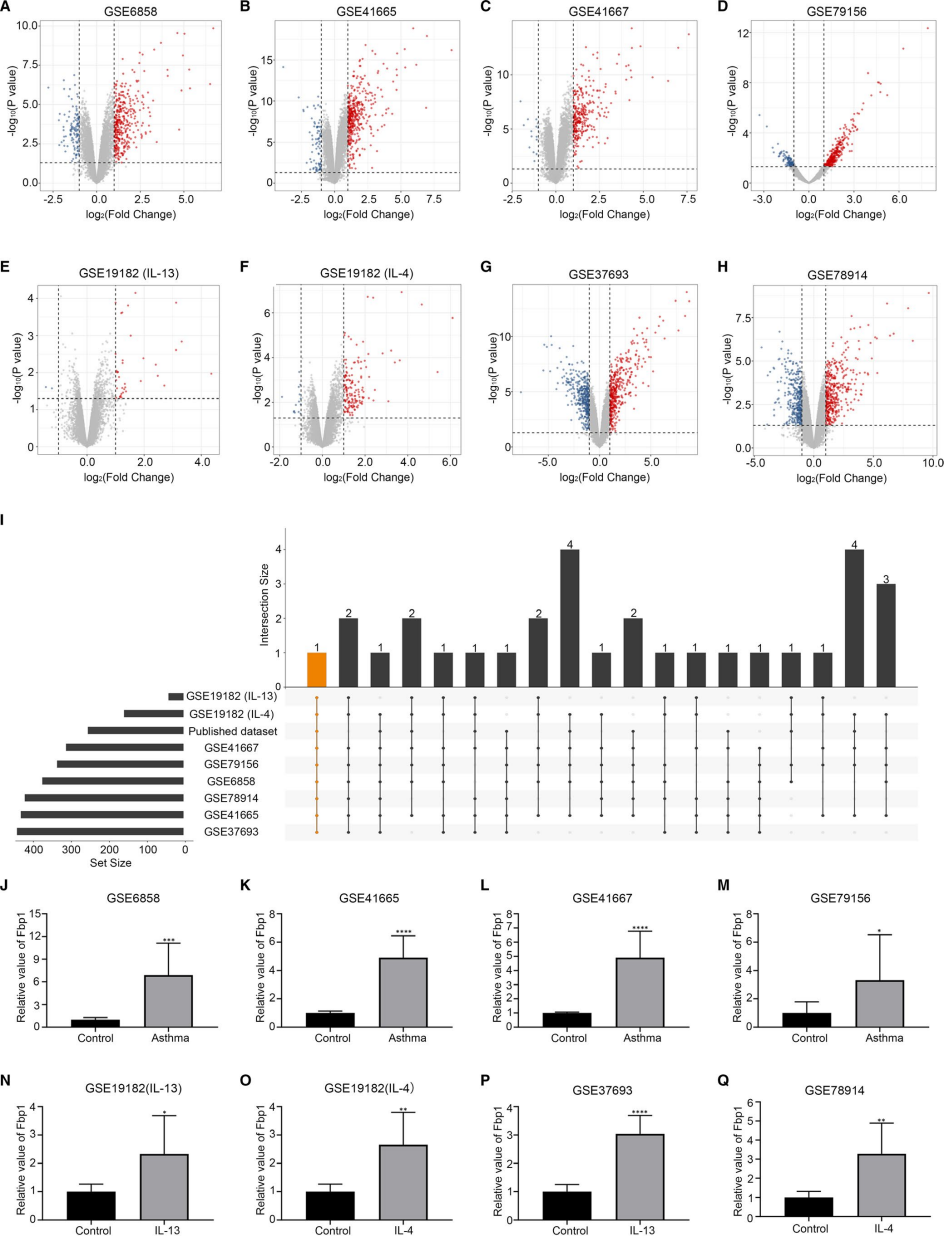
Fbp1 is overexpressed in a murine model of asthma and IL‐4 or IL‐13 induced bronchial epithelial cells based on bioinformatics analyses. Volcano plots of GSE6858 (A), GSE41665 (B), GSE41667 (C) and GSE79156 (D) showing comparative mRNA gene expression in asthmatic and control groups. Red and blue dots represent significantly up‐regulated and down‐regulated genes, respectively. Volcano plots of GSE19182 (IL‐13 stimulated group) (E), GSE19182 (IL‐4 stimulated group) (F), GSE37693 (G) and GSE78914 (H) showing comparative mRNA gene expression in IL‐4 or IL‐13 induced bronchial epithelial cells and control cells. I, UpSet diagram showing overlapping genes from the up‐regulated genes in the nine data sets (horizontal bar). Each column represents shared genes among these data sets (linked dots). The orange column represents the gene all of the nine data sets shared. The relative expression of Fbp1 in GSE6858 (J), GSE41665 (K), GSE41667 (L) and GSE79156 (M). The relative expression of Fbp1 in GSE19182 (IL‐13 stimulated group) (N), GSE19182 (IL‐4 stimulated group) (O), GSE37693 (P) and GSE78914 (Q). *P < .05, **P < .01, ***P < .001, ****P < .0001

A bioinformatics approach was used to identify the DEGs in the data sets, which were visualized as volcano plots (Figure 1A‐H). We then considered the intersection of the up‐regulated genes from the nine data sets using UpSetR of the R package and a single gene, Fbp1, was selected (Figure 1I). As shown in Figure 1J‐Q, the Fbp1 relative values were substantially higher in asthmatic mice than in control mice (Figure 1J‐M). Furthermore, the Fbp1 relative values were significantly increased in IL‐4‐stimulated or IL‐13‐stimulated bronchial epithelial cells compared to those in control cells (Figure 1N‐Q). These results suggest Fbp1 expression is increased in asthma and may play an important role in disease progression.

### 3.2 Fbp1 was overexpressed in a murine model of asthma and IL‐4‐stimulated or IL‐13‐stimulated bronchial epithelial cells

**FIGURE 2:**
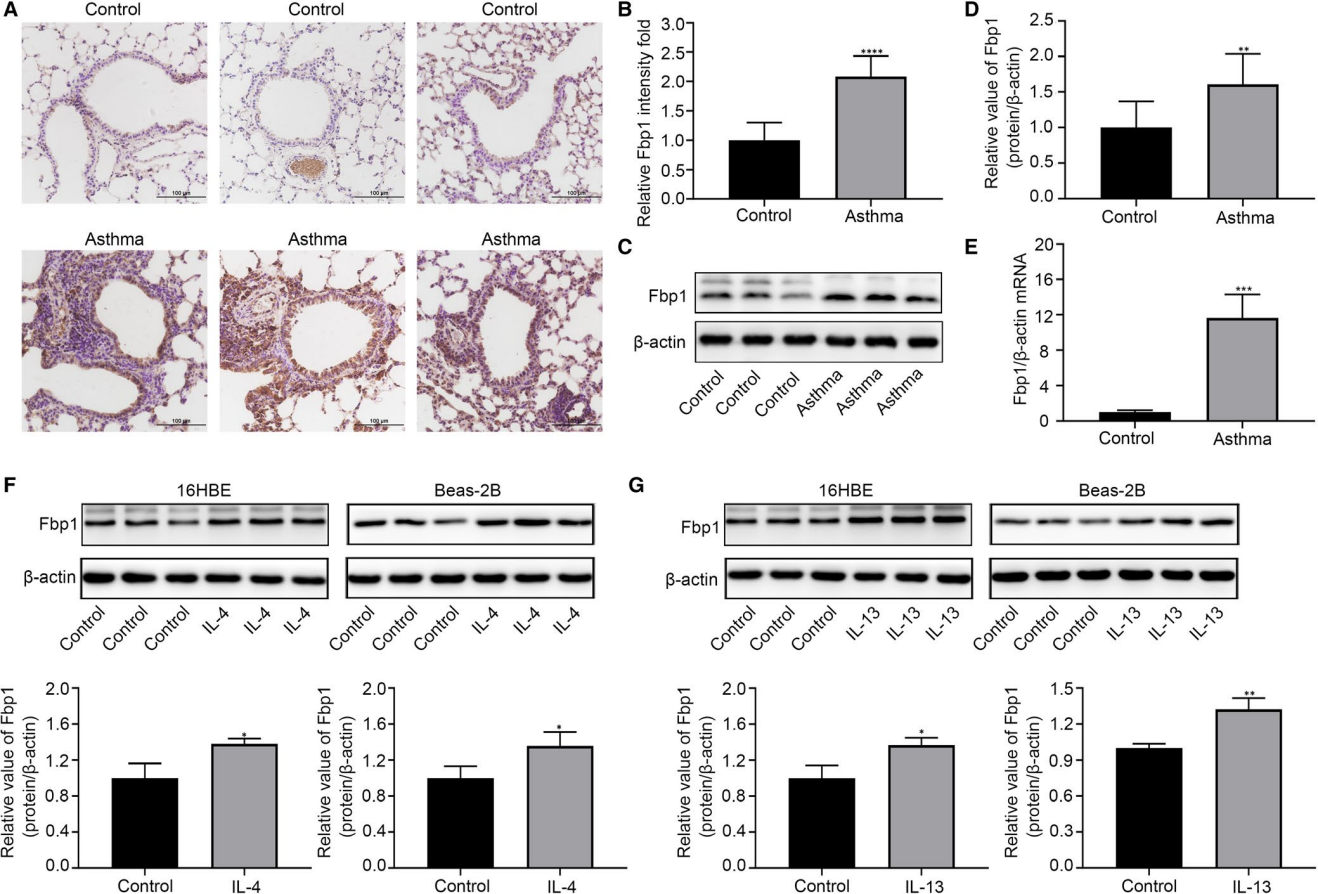
The expression of Fbp1 is increased in the bronchial epithelial cells of asthmatic mice and 16HBE and Beas‐2B cells after IL‐4 or IL‐13 treatment. A, Immunohistochemistry of Fbp1 expression in lung tissues of asthmatic and control mice (magnification ×200). B, Semi‐quantitative analysis of Fbp1 expression in the bronchial epithelial cells of lung tissues based on immunohistochemistry. C, D, Protein expression Fbp1 in lung tissues of asthmatic and control mice. E, Relative mRNA expression of Fbp1 in lung tissues of asthmatic and control mice. Protein expression of Fbp1 in 16HBE and Beas‐2B cells after treatment with (F) IL‐4 and (G) IL‐13. *P < .05, **P < .01, ***P < .001, ****P < .0001

To verify the bioinformatics analyses results, we first established an animal model of asthma (Figure S1A‐D). While Fbp1 expression was observed in bronchial epithelial cells of both asthmatic and control mice, immunohistochemical analyses revealed that the expression of Fbp1 in asthmatic epithelial cells was significantly higher than that in the control group (Figure 2A,B). Moreover, Fbp1 mRNA and protein levels exhibited similar changes (Figure 2C‐E).

IL‐4 and IL‐13 were then used individually to stimulate 16HBE and Beas‐2B cells. Consistent with p‐Stat6 being specifically activated by IL‐4 and IL‐13,^31^, ^32^ Stat6 phosphorylation was observed following incubation of 16HBE and Beas‐2B cells with IL‐4 or IL‐13 (Figure S1E,F). Accordingly, both cytokines were considered active components in this experiment. After treatment of 16HBE and Beas‐2B cells with IL‐4 or IL‐13, the expression levels of epithelial‐mesenchymal transition (EMT) markers were unchanged or only slightly changed (Figure S1G‐J). Western blot analysis demonstrated that the protein levels of Fbp1 in IL‐4‐stimulated or IL‐13‐stimulated 16HBE and Beas‐2B cells were higher than those in control cells (Figure 2F‐G). Overall, these data indicate that the mRNA and protein expression of Fbp1 may be up‐regulated in bronchial epithelial cells in asthma.

### 3.3 Fbp1 induced cell apoptosis and G2/M arrest in vitro

**FIGURE 3:**
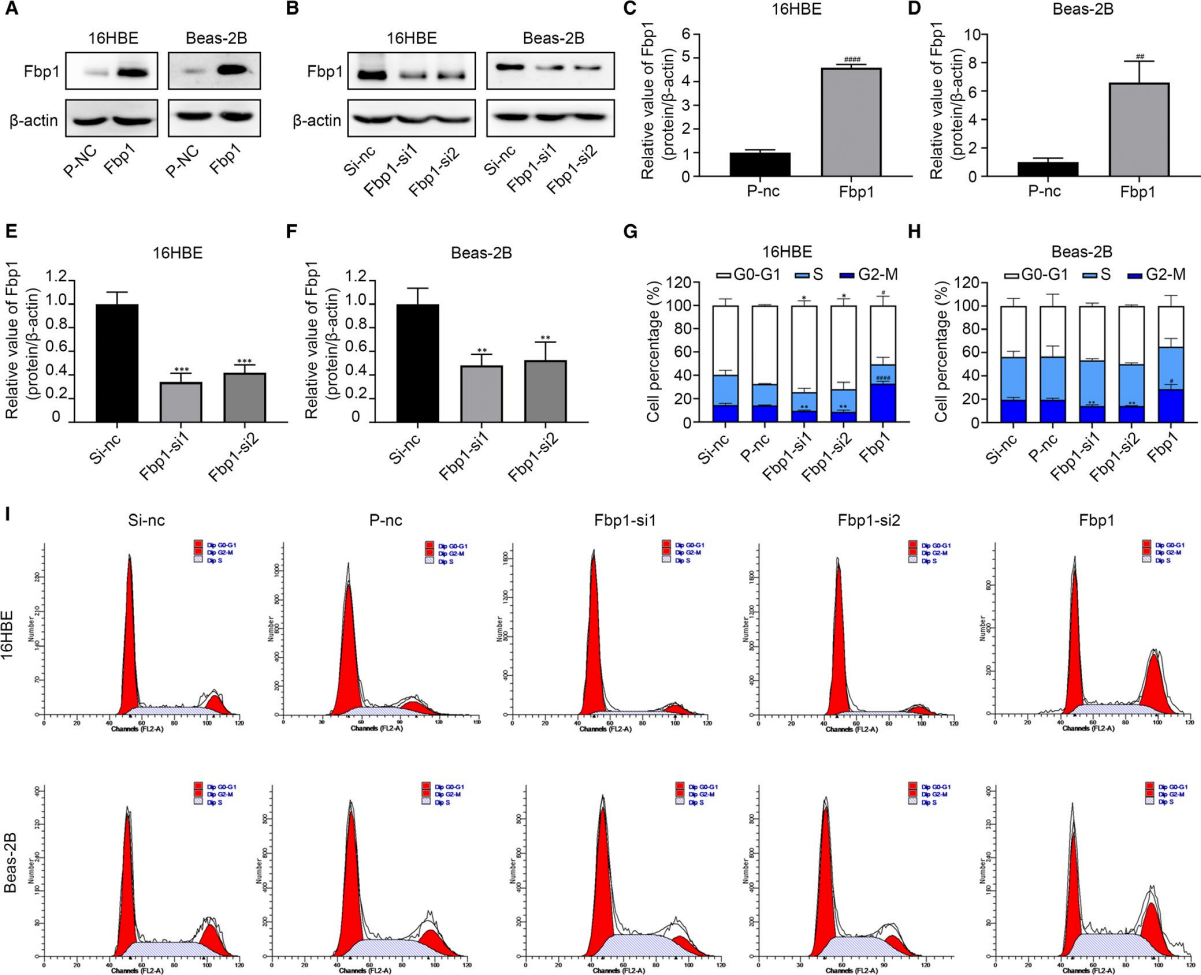
Effects of Fbp1 on the cell cycle of 16HBE and Beas‐2B cells. A, C, D, Protein expression levels of Fbp1 after overexpression plasmid transfection. B, E, F, Protein expression levels of Fbp1 after Fbp1 siRNA1 and Fbp1 siRNA2 transfection. G‐I, Each phase percentage of the cell cycle was determined by flow cytometry. *P < .05, **P < .01, ***P < .001 as compared to the Si‐nc group; ^#^ *P* < .05, ^##^ *P* < .01, ^####^ *P* < .0001 as compared to the P‐nc group

**FIGURE 4:**
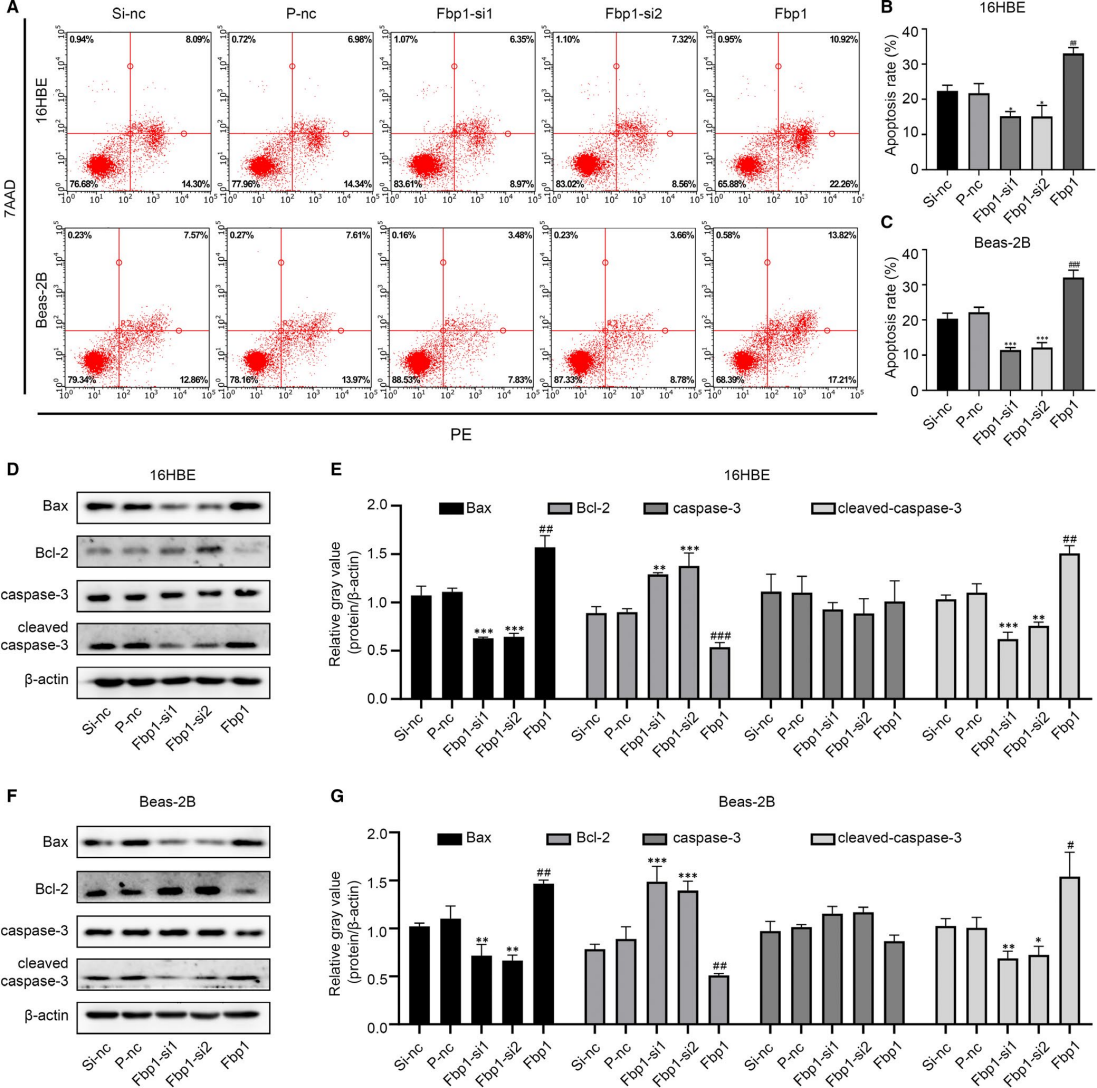
Effects of Fbp1 on cell apoptosis in 16HBE and Beas‐2B cells. A‐C, Apoptosis of 16HBE and Beas‐2B cells detected by flow cytometry after knock‐down or overexpression of Fbp1. D‐G, Protein expression levels of Bax, Bcl‐2, caspase‐3 and cleaved‐caspase‐3 in the different groups. *P < .05, **P < .01, ***P < .001 as compared to the Si‐nc group; ^#^ *P* < .05, ^##^ *P* < .01, ^###^ *P* < .001 as compared to the P‐nc group

To investigate the biological function and mechanism of action of Fbp1 in epithelial cells, 16HBE and Beas‐2B cells were transfected with Fbp1 siRNA and Fbp1 plasmid. The protein levels of Fbp1 were significantly decreased after transfection with Fbp1 siRNA1 and Fbp1 siRNA2 and increased after transfection with Fbp1 plasmid (Figure 3A‐F). Knock‐down of Fbp1 prior to IL‐13 treatment resulted in decreased apoptosis, while overexpression of Fbp1 led to increased apoptosis (Figure 4A‐C). Western blot analysis demonstrated that Fbp1 silencing reduced the levels of Bax and cleaved‐caspase‐3 and increased the levels of Bcl‐2 in 16HBE and Beas‐2B cells; however, the level of caspase‐3 did not differ between the Fbp1‐knock‐down cells and Si‐nc‐transfected cells (Figure 4D‐G). In addition, overexpression of Fbp1 led to increased levels of Bax and cleaved‐caspase‐3 and reduced levels of Bcl‐2 in both 16HBE and Beas‐2B cells. The level of caspase‐3 did not change in either 16HBE or Beas‐2B cells (Figure 4D‐G). These results demonstrate that Fbp1 was able to induce apoptosis in 16HBE and Beas‐2B cells.

Cell cycle arrest is closely associated with apoptosis, and blockage of cell cycle progression may result in apoptosis. To elucidate the cause of bronchial epithelial cell apoptosis, flow cytometry was used to measure the effect of Fbp1 on cell cycle transition. Fbp1 knock‐down significantly reduced the proportion of cells in the G2/M phase for both cell lines, and Fbp1 overexpression significantly increased the proportion of cells in the G2/M phase (Figure 3G‐I). Taken together, Fbp1 may induce G2/M phase arrest followed by apoptosis.

### 3.4 Fbp1 aggravated oxidative stress in vitro

**FIGURE 5:**
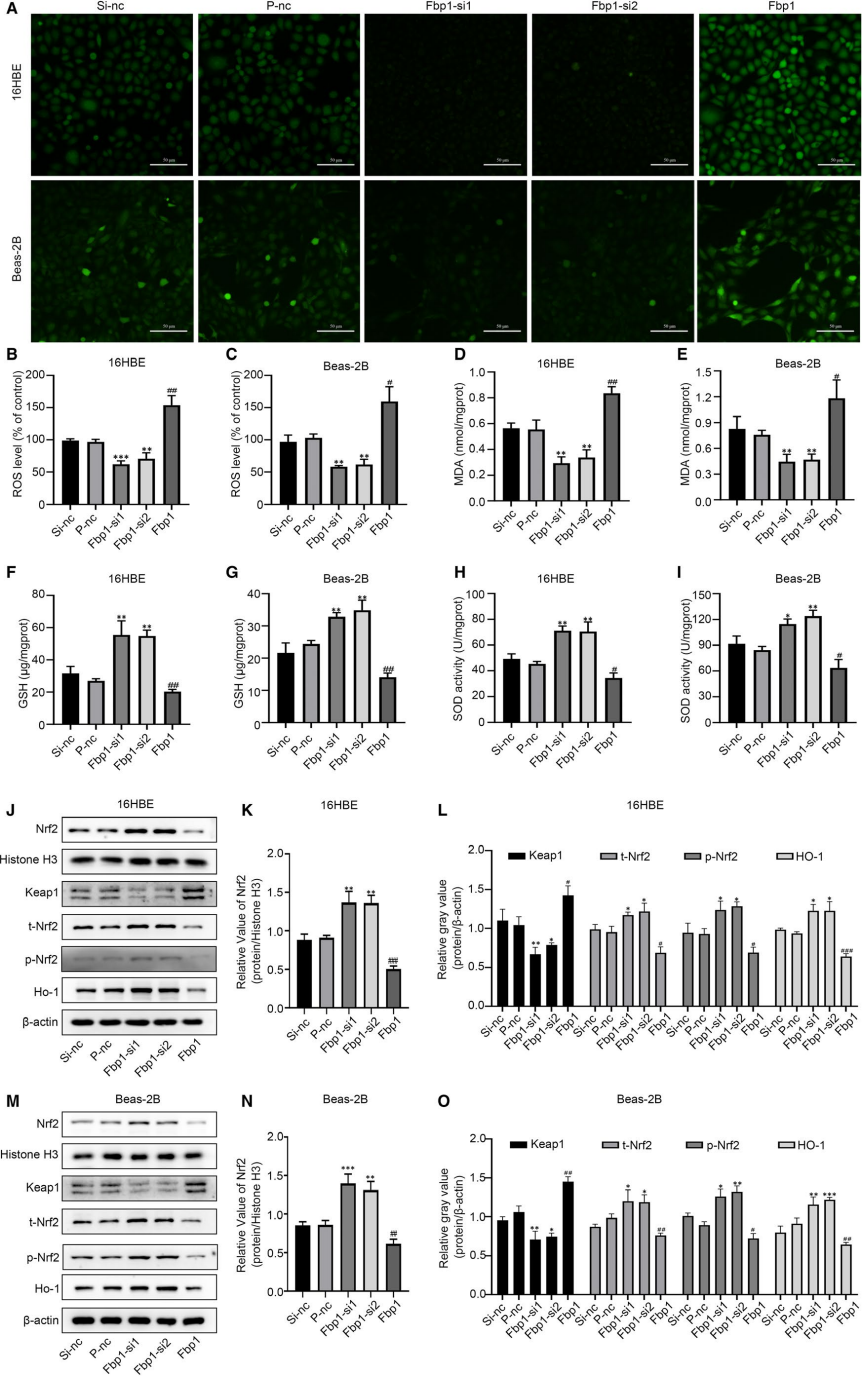
Effects of Fbp1 on oxidative stress and the Nrf2 pathway in vitro. A, ROS levels detected by fluorescence microscopy after knock‐down or overexpression of Fbp1 (magnification ×200). B, C, ROS levels detected by Multi‐Mode Microplate Reader. D, E, malondialdehyde (MDA) content, (F, G) glutathione (GSH) content, and (H, I) superoxide dismutase (SOD) activity in the different groups. J‐O, Protein expression levels of nuclear Nrf2, Keap1, total‐Nrf2, p‐Nrf2 and HO‐1 in the different groups. *P < .05, **P < .01, ***P < .001 as compared to the Si‐nc group; ^#^ *P* < .05, ^##^ *P* < .01, ^###^ *P* < .001 as compared to the P‐nc group

We then measured the effect of Fbp1 on ROS levels in IL‐13‐stimulated 16HBE and Beas‐2B cells. Fbp1 knock‐down reduced the levels of ROS in the 16HBE and Beas‐2B cells based on the amount of green fluorescence detected by fluorescence microscopy and the Multi‐Mode Microplate Reader, while Fbp1 overexpression increased the ROS levels (Figure 5A‐C).

We then evaluated the levels of GSH, SOD and MDA, which are closely associated with oxidative stress. As indicated in Figure 4D‐I, the levels of GSH and SOD were significantly increased in the Fbp1‐siRNA‐transfected group compared to those in the control group, while the level of MDA was significantly decreased. In contrast, Fbp1 overexpression significantly reduced GSH and SOD levels but resulted in elevated MDA levels. Taken together, the data suggest that the Fbp1 could aggravate oxidative stress in 16HBE and Beas‐2B cells.

### 3.5 Fbp1 induced apoptosis and aggravated oxidative stress by inhibiting the Nrf2 pathway

To investigate the mechanism underlying the effect of Fbp1 on apoptosis and oxidative stress, we analysed the levels of Keap1, Nrf2 and HO‐1 by Western blotting. The protein levels of total‐Nrf2, p‐Nrf2, HO‐1 and nuclear Nrf2 were significantly higher in the knock‐down groups than in the control group, whereas the protein levels of Keap1 were significantly reduced (Figure 5J‐O). In comparison, the expression of total‐Nrf2, p‐Nrf2, HO‐1 and nuclear Nrf2 was significantly lower in the overexpression group than in the control group, whereas the expression of Keap1 was significantly elevated (Figure 5J‐O). These results indicate that Fbp1 enhanced oxidative stress‐induced apoptosis by suppressing the Keap1/Nrf2/HO‐1 signalling pathway.

### 3.6 Aggravation of oxidative stress‐induced apoptosis by Fbp1 was partially reversed by Nrf2 inhibitor ML385

**FIGURE 6:**
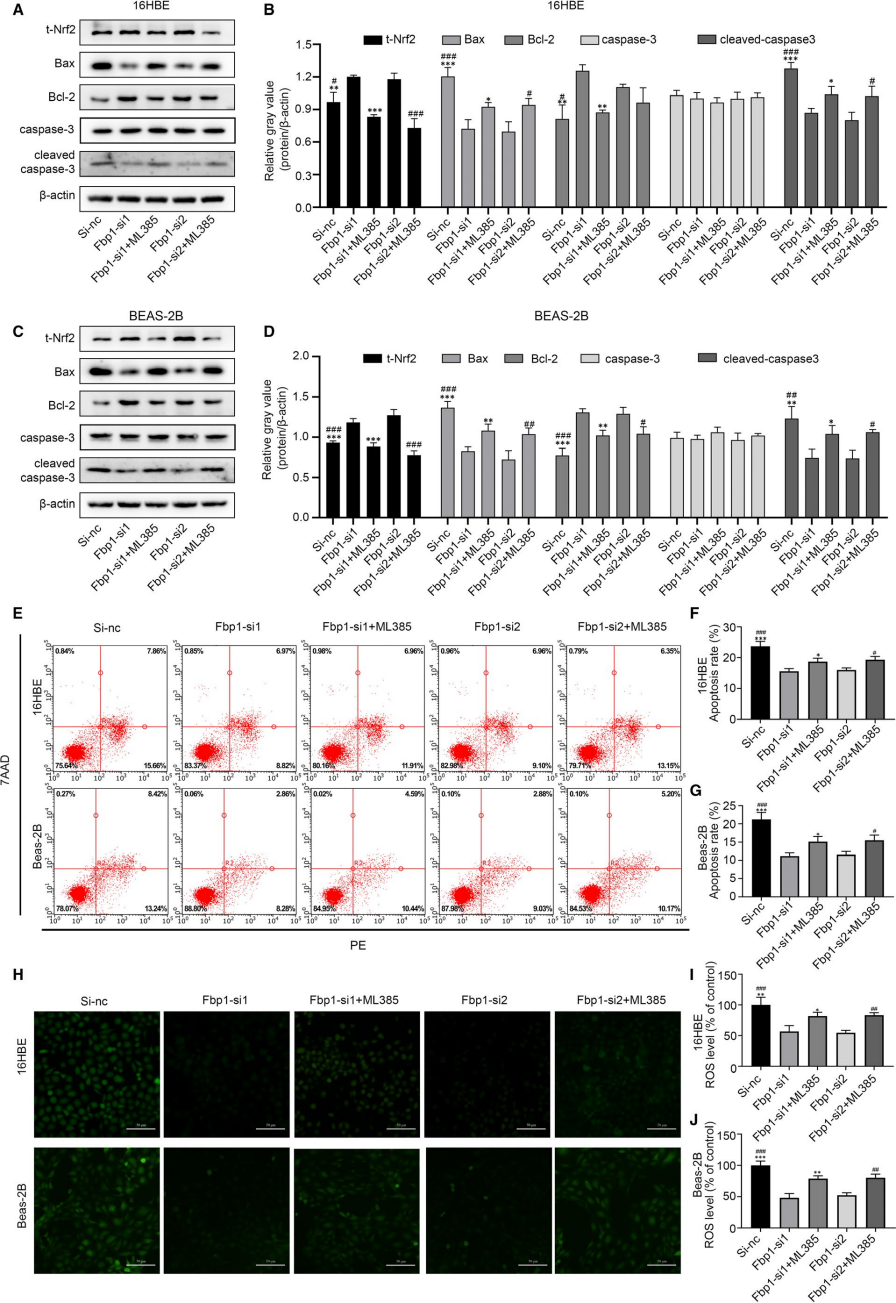
Nrf2 inhibitor, ML385, can partially reverse oxidative stress‐induced apoptosis caused by Fbp1‐silencing. A‐D, Protein expression levels of total‐Nrf2, Bax, Bcl‐2, caspase‐3, and cleaved‐caspase‐3 after Fbp1 knock‐down simultaneously with ML385 or DMSO in 16HBE and Beas‐2B cells. E‐G, Apoptosis rate assessed by flow cytometry in the different groups. ROS measured by (H) fluorescence microscope (magnification ×200) and (I, J) Multi‐Mode Microplate Reader. *P < .05, **P < .01, ***P < .001 as compared to the Fbp1‐si1 group; ^#^ *P* < .05, ^##^ *P* < .01, ^###^ *P* < .001 as compared to the Fbp1‐si2 group

To explore whether the Nrf2 pathway plays a role in the mechanism by which Fbp1 exacerbates apoptosis induced by oxidative stress, we administered the Nrf2 inhibitor ML385 during transfection of the 16HBE and Beas‐2B cells. As shown in Figure 6A‐D, the expression levels of Nrf2 were markedly decreased after the addition of ML385. Knock‐down of Fbp1 resulted in decreased Bax and cleaved‐caspase‐3 expression in 16HBE and Beas‐2B cells and increased Bcl‐2 expression. However, ML385 treatment during transfection partially reversed the effect of Fbp1 knock‐down on the expression of Bax and cleaved‐caspase‐3 in both cell lines. Meanwhile, Bcl‐2 levels were significantly lower in the ML385 treatment groups than in the knock‐down groups, except for siRNA2 in 16HBE cells (Figure 6A‐D). These results suggest that suppressing Fbp1 alleviated apoptosis through the Nrf2 signalling pathway.

We then assessed the apoptosis rate after ML385 treatment in Fbp1‐siRNA‐transfected 16HBE and Beas‐2B cells using flow cytometry. The down‐regulation of apoptosis was greater in the ML385‐treated group than in the untreated knock‐down group, whereas apoptosis was lower than that in the negative control group (Figure 6E‐G). This was consistent with the change in apoptosis‐related protein levels. In addition, knock‐down of Fbp1 alleviated ROS levels in both 16HBE and Beas‐2B cells, but ML385 treatment partially reversed the changes in ROS levels after Fbp1‐siRNA transfection (Figure 6H‐J). These results suggest that the Nrf2 inhibitor ML385 could partially reverse the aggravation of apoptosis caused by Fbp1‐induced oxidative stress.

## 4 DISCUSSION

Allergic asthma is a widespread chronic respiratory disease that results in significant morbidity and mortality worldwide and has attracted substantial attention from the scientific community. However, in order to develop more effective treatment strategies, there is a need for improved understanding of the underlying mechanisms of asthma. Towards this end, we retrieved four data sets of a murine model of ovalbumin‐induced asthma and four data sets of IL‐4‐stimulated or IL‐13‐stimulated bronchial epithelial cells from the GEO database as well as one data set from a previously published murine asthma model. These data sets were then used to analyse the DEGs in asthma. Core genes were screened by the intersection of the nine data sets, and a single gene, Fbp1, was ultimately selected.

Fbp1 is a rate‐limiting enzyme in gluconeogenesis and a key member of the Fbp family. Previous studies on Fbp1 have mainly focused on its function in glucose metabolism. Fbp1 maintains the balance between fructose‐1,6‐diphosphate and fructose‐6‐phosphate during glycolysis.^33^ Fbp1 deficiency results in ketotic hypoglycaemia and lactic acidosis.^34^ Notably, Fbp1 inhibitors have been reported as effective in the management of type 2 diabetes.^9^ Recent research on Fbp1 has focused on its role in cancer. For instance, Fbp1 has been reported to play an anti‐oncogenic role, inhibiting cell proliferation, invasion, migration and EMT in various cancers, including pancreatic cancer,^35^ breast cancer^36^ and gastric cancer.^37^ However, the expression and function of Fbp1 have not been investigated in allergic diseases such as asthma.

In the current study, we observed Fbp1 expression in bronchial epithelial cells localized in the lung. We first determined that Fbp1 was significantly up‐regulated in both asthmatic mouse lungs and bronchial epithelial cells incubated with IL‐4 or IL‐13. However, this contradicts previous studies reporting that EMT plays an important role in asthma. Fbp1 and EMT exhibit bidirectional inhibition in hepatocellular carcinoma; overexpression of FBP1 can inhibit EMT, whereas EMT can inhibit the expression of FBP1.^11^ Based on this point, Fbp1 would be expected to be expressed at low levels, which is in contrast to our results. Therefore, we evaluated the changes in EMT stimulated by IL‐4 or IL‐13. After incubation with IL‐4 or IL‐13, we found only slight changes in EMT in 16HBE and Beas‐2B cells. Burgess et al^38^ reported similar results in IL‐13‐induced Beas‐2B cells and Ji et al^39^ found that IL‐4 did not induce EMT in 16HBE cells. Thus, EMT may not be important in IL‐4‐ or IL‐13‐induced 16HBE and Beas‐2B cells. This is consistent with our results suggesting that Fbp1 may be involved in the pathogenesis of asthma and that its elevated level in epithelial cells following ovalbumin challenge or IL‐4 or IL‐13 treatment may be a detrimental mechanism inducing injury in bronchial epithelial cells.

Apoptosis is a form of programmed cell death that plays an essential role in lung disease, such as asthma. Elevated levels of apoptosis in bronchial epithelial cells have been reported in patients with childhood asthma.^40^, ^41^ Therefore, it is important to investigate the potential mechanism of apoptosis in asthma. Some studies have reported that Fbp1 can promote apoptosis in certain cancers. For example, overexpression of Fbp1 in cholangiocarcinoma enhances apoptosis.^14^ In addition, loss of Fbp1 reportedly promotes apoptosis‐resistance in cancer stem‐like cells and enhances tumorigenesis.^42^ Consistent with this, our results demonstrated that Fbp1 silencing in 16HBE and Beas‐2B cells reduced apoptosis, whereas Fbp1 overexpression promoted apoptosis. As it has been reported that cell cycle arrest can be an important initiator of cell apoptosis,^43^ we carried out cell cycle analyses and found that Fbp1 knock‐down significantly decreased the number of G2/M cells, whereas Fbp1 overexpression increased the number of cells in the G2/M phase. Considering these results, we conclude that Fbp1 is a pro‐apoptotic molecule in IL‐13‐stimulated bronchial epithelial cells and that this may be attributed to cell cycle arrest in the G2/M phase, which blocks cell recovery and division.

We then investigated oxidative stress as a potential mechanism of Fbp1‐induced apoptosis. It is well‐known that cell cycle arrest and apoptosis can be induced by excessive oxidative stress.^44^, ^45^, ^46^ It is also known that oxidative stress plays a key role in the development of chronic diseases, including diabetes, cardiovascular disorders, cancer and asthma.^47^ Allergic asthma is a respiratory disorder involving allergic reactions, airway inflammation and apoptosis. Excessive oxidative stress in asthma results in exacerbated bronchospasms, aggravates airway inflammation and enhances apoptosis that may worsen lung damage.^48^, ^49^ Notably, it has been reported that natural antioxidants can alleviate the pathological progression of asthma by suppressing oxidative stress.^50^, ^51^ It has been reported that Fbp1 exhibits pro‐oxidative effects on basal‐like breast cancer and that Fbp1‐expressing cells show a marked increase in ROS levels.^15^ Forced Fbp1 expression also restores ROS generation in cancer stem‐like cells.^42^ In the current study, Fbp1 knock‐down attenuated ROS and MDA levels and increased the expression of antioxidant enzymes SOD and GSH. Meanwhile, Fbp1 overexpression increased ROS and MDA levels and decreased SOD and GSH levels. These results indicate that Fbp1 induced abnormal oxidative stress accumulation, which could subsequently lead to apoptosis and cell arrest. Thus, Fbp1 may be a potential target for improving asthma treatment.

Apoptosis related to oxidative stress is often accompanied by Nrf2 pathway inactivation.^52^ Nrf2, a protective transcription factor closely associated with asthma, is associated with antioxidant and anti‐apoptotic activity. In an animal model of asthma, Nrf2 knockout not only resulted in increased levels of eosinophils and neutrophils in BALF and lung tissues, which generate more ROS, but also aggravated AHR, epithelial cell apoptosis and goblet cell hyperplasia.^53^ Under normal conditions, Nrf2 exists in an inactive state by binding to the inhibitory protein Keap1. In response to stress, such as oxidative stress, endoplasmic reticulum stress or hyperglycaemia, Nrf2 is phosphorylated and released from Keap1 and then translocates to the nucleus where it promotes the expression of antioxidant genes such as HO‐1.^54^, ^55^, ^56^, ^57^ However, the relationship between the Nrf2 pathway and Fbp1 remains unclear. In an attempt to gain further insight into the molecular processes underlying asthma, we evaluated the protein expression of this pathway. Fbp1 knock‐down by siRNA increased protein levels of total Nrf2, p‐Nrf2, nuclear Nrf2 and HO‐1 and decreased levels of Keap1. In contrast, Fbp1 overexpression by Fbp1 plasmid decreased the expression of total Nrf2, p‐Nrf2, nuclear Nrf2 and HO‐1 and increased the expression of Keap1. This suggests that Fbp1 induced apoptosis and oxidative stress in IL‐13‐stimulated bronchial epithelial cells via suppression of the Keap1/Nrf2/HO‐1 pathway. To further resolve the details of this mechanism, the Nrf2 inhibitor ML385 was used to investigate whether Fbp1 performed pro‐apoptosis and pro‐oxidative functions through the Nrf2 pathway. We found that co‐treatment of cells with ML385 and Fbp1 siRNA partially reversed the decreased apoptosis and oxidative stress induced by Fbp1 knock‐down. Therefore, the Nrf2 pathway appears to be integral to Fbp1‐related oxidative stress‐induced apoptosis. The potential mechanisms remain to be further explored.

In conclusion, we showed that Fbp1 expression was increased in the bronchial epithelial cells of a murine model of allergic asthma. Fbp1 was also up‐regulated by IL‐4 or IL‐13 in human bronchial epithelial cells. Furthermore, Fbp1 silencing reduced oxidative stress‐induced apoptosis by activating the Nrf2 pathway and Fbp1‐overexpression aggravated oxidative stress‐induced apoptosis by suppressing this pathway. Moreover, Nrf2 inhibitor ML385 could partially reverse the anti‐oxidative and anti‐apoptosis function induced by Fbp1 knock‐down. Collectively, our results suggest that Fbp1 may be a potential therapeutic target related to bronchial epithelial cell apoptosis and oxidative stress in asthma.

## CONFLICT OF INTEREST

There are no potential conflicts of interest to declare.

## AUTHOR CONTRIBUTION

**Jiapeng Hu:** Data curation (lead); Methodology (lead); Software (lead); Visualization (lead); Writing‐original draft (lead). **Jia Wang:** Data curation (supporting); Methodology (supporting). **Chunlu Li:** Data curation (supporting); Visualization (supporting). **Yunxiao Shang:** Funding acquisition (lead); Project administration (lead); Resources (lead); Supervision (lead); Writing‐review & editing (lead).

## Supporting information

Figure S1

Supplementary Material

## Data Availability

All data that support the findings of this study are available from the corresponding author upon reasonable request.
