# Supplementary figures and images for: Fructose‐1,6‐bisphosphatase aggravates oxidative stress‐induced apoptosis in asthma by suppressing the Nrf2 pathway
Source: J Cell Mol Med. 2021 May 7;25(11):5001–14. doi: 10.1111/jcmm.16439 (PMC8178285; doi:10.1111/jcmm.16439)

**A**

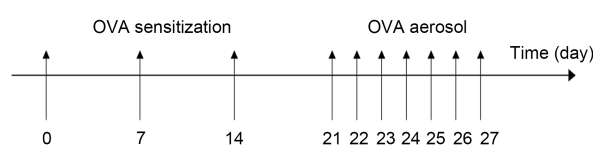

**B**

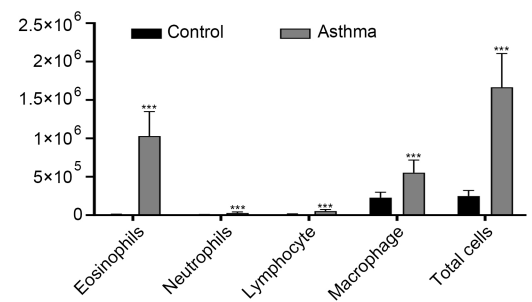

**C**

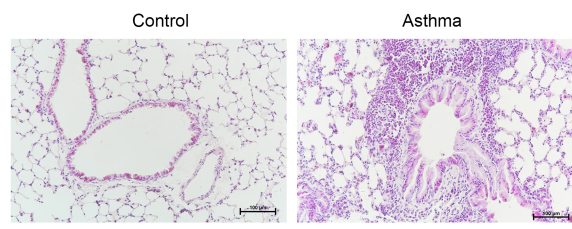

**D**

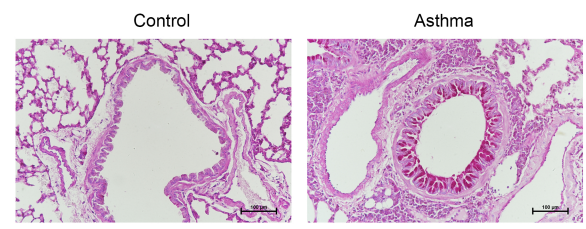

**E**

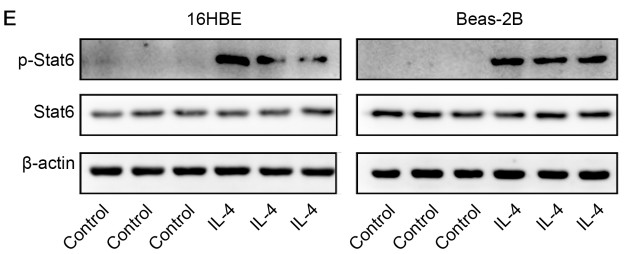

**F**

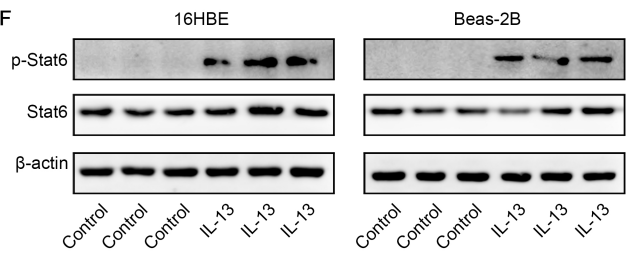

**G**

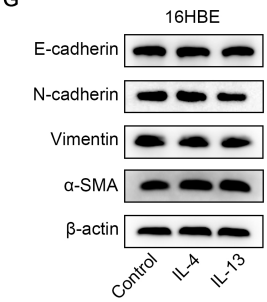

**H**

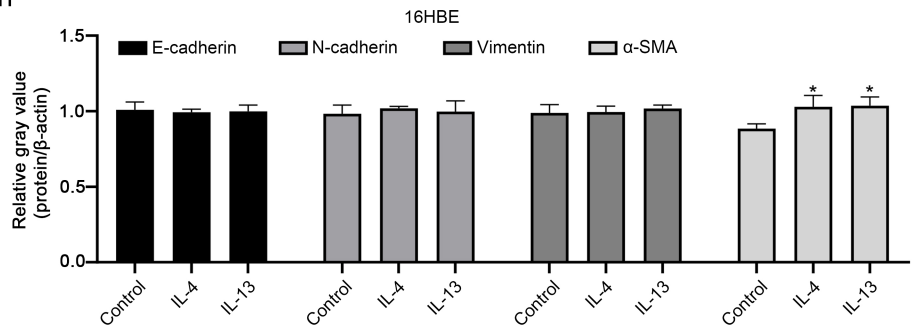

**I**

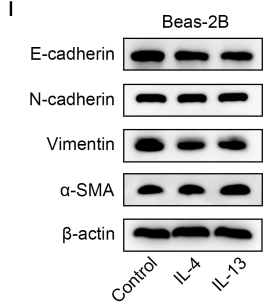

**J**

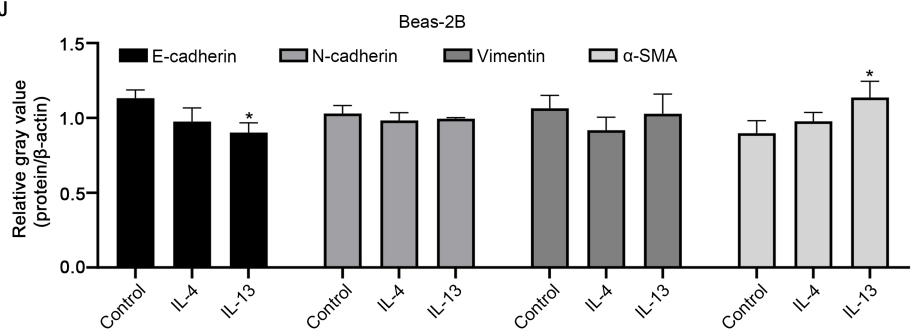

Supplement: Supplementary file 1 — Figure S1 [file JCMM-25-5001-s002.pdf]
