# Supplementary material for: Fructose‐1,6‐bisphosphatase aggravates oxidative stress‐induced apoptosis in asthma by suppressing the Nrf2 pathway
Source: J Cell Mol Med. 2021 May 7;25(11):5001–14. doi: 10.1111/jcmm.16439 (PMC8178285; doi:10.1111/jcmm.16439)
Supplement: Supplementary file 2 — Supplementary Material [file JCMM-25-5001-s001.docx]

**Supporting Information**

Figure S1. Establishment of an ovalbumin-induced murine model of asthma and IL-4- or IL-13-stimulated bronchial cells. (A) The protocol for creating the murine asthma model. (B) Total and differentiated inflammatory cell counts in BALF from asthmatic and control mice. (C) HE and (D) PAS staining of lung tissue (magnification ×200). Protein expression levels of p-Stat6 and Stat6 in (E) IL-4-stimulated and IL-(F) 13-stimulated 16HBE and Beas-2B cells. (G, H) Protein expression levels of E-cadherin, N-cadherin, vimentin, and α-SMA after treatment with IL-4 or IL-13 in 16HBE cell. (I, J) Protein expression levels of E-cadherin, N-cadherin, vimentin, and α-SMA after treatment with IL-4 or IL-13 in Beas-2B cell. *P<0.05, ***P<0.001.
